# Imaging of Neuroendocrine Prostatic Carcinoma

**DOI:** 10.3390/cancers13225765

**Published:** 2021-11-17

**Authors:** Ahmed Taher, Corey T. Jensen, Sireesha Yedururi, Devaki Shilpa Surasi, Silvana C. Faria, Tharakeshwar K. Bathala, Bilal Mujtaba, Priya Bhosale, Nicolaus Wagner-Bartak, Ajaykumar C. Morani

**Affiliations:** 1 Department of Diagnostic Radiology, The University of Texas MD Anderson Cancer Center, 1515 Holocombe Blvd., Houston, TX 77030, USA; Ahmedramadantawfik@gmail.com (A.T.); CJensen@mdanderson.org (C.T.J.); SYedururi@mdanderson.org (S.Y.); Scfaria@mdanderson.org (S.C.F.); TKBathala@mdanderson.org (T.K.B.); BMujtaba@mdanderson.org (B.M.); Priya.Bhosale@mdanderson.org (P.B.); NWagner@mdanderson.org (N.W.-B.); 2 Department of Nuclear Medicine, The University of Texas MD Anderson Cancer Center, 1515 Holcombe Blvd., Houston, TX 77030, USA; dssurasi@mdanderson.org

**Keywords:** prostate, neuroendocrine, prostatic neuroendocrine carcinoma, small cell carcinoma of prostate

## Abstract

**Simple Summary:**

Neuroendocrine prostate cancer (NEPC) is an aggressive type of prostate cancer with a very high potential for distant metastatic spread in the body. It is associated with poor survival in comparison to the usual adenocarcinoma type of prostate cancer. Although it can arise de novo, NEPC much more commonly occurs as a mechanism of resistance during treatment for usual type prostatic adenocarcinoma, the latter is also called as castration-resistant prostate cancer (CRPC). The incidence of NEPC increases after hormonal therapy and they represent a challenge, both in the radiological and pathological diagnosis, as well as in the clinical management. This article provides a comprehensive imaging review of prostatic neuroendocrine tumors.

**Abstract:**

Neuroendocrine prostate cancer (NEPC) is an aggressive subtype of prostate cancer that typically has a high metastatic potential and poor prognosis in comparison to the adenocarcinoma subtype. Although it can arise de novo, NEPC much more commonly occurs as a mechanism of treatment resistance during therapy for conventional prostatic adenocarcinoma, the latter is also termed as castration-resistant prostate cancer (CRPC). The incidence of NEPC increases after hormonal therapy and they represent a challenge, both in the radiological and pathological diagnosis, as well as in the clinical management. This article provides a comprehensive imaging review of prostatic neuroendocrine tumors.

## 1. Introduction

**Figure 1:**
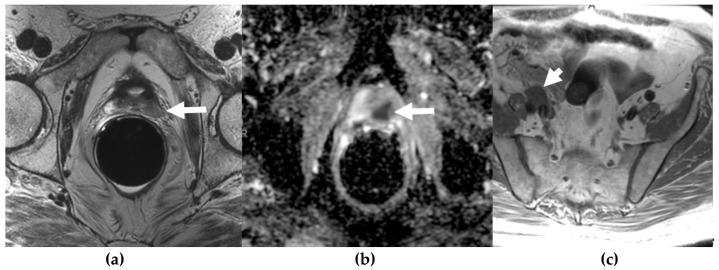
A 60-year-old man with lower retroperitoneal lymphadenopathy on PET-CT, found during work-up for dermatomyositis and abnormal digital rectal exam. T2W (**a**) and ADC (**b**) MR images through the prostate show a hypointense mass (long arrow) with restricted diffusion in the left posterolateral peripheral zone of the prostatic apex. Large field of view axial T1W MR image (**c**) of the pelvis shows right external iliac lymphadenopathy (short arrow). This was biopsy-proven metastatic small cell carcinoma of the prostate.

Prostate cancer is the most common non-cutaneous cancer in men worldwide. The year 2020 estimates for prostate cancer are about 191,930 new cases in the United States and 1,414,259 worldwide and it is the second leading cause of cancer death in men, behind lung cancer [1,2]. Neuroendocrine prostate cancer (NEPC) represents an aggressive subtype of prostate cancer, accounting for 0.5–2% of all prostate cancers and typically has a high metastatic potential and poor prognosis [3]. It can arise de novo (Figure 1), but much more commonly occurs as a mechanism of treatment resistance during therapy for conventional prostatic adenocarcinoma, when they are also termed castration-resistant prostate cancers (CRPC) [4]. Thus, the incidence of NEPCs increases after hormonal therapy and these are thought to arise from lineage plasticity induced by androgen receptor-targeted therapy [5]. They represent a challenge in the radiological and pathological diagnosis, as well as in the clinical management of the patients with limited therapies and very poor prognosis. Our objective is to offer an overview of their radiological characteristics with radiopathologic correlations and illustrations.

## 2. Pathologic Classification and Genetic Alterations

The main function of the prostate gland is to produce an alkaline fluid, one of the components of semen, which nourishes and protects sperm. Glands formed from epithelial cells produce these secretions. Histologically, the prostate gland includes two main types of epithelial cells: basal cells and luminal cells, which can be readily identified using light microscopy (LM). Neuroendocrine cells represent a third cell type that constitute 1% or less of the total prostatic cell population and are found scattered between the basal and luminal cells [6]. Morphologically, they are of two types: “open” cells that are flask-shaped with apical processes towards the lumen and “closed” cells that interdigitate with secretory cells and have long dendritic processes [7]. They do not express prostate-specific antigen (PSA), which is an epithelial differentiation marker, rather they express neuroendocrine markers, including neuron-specific enolase (NSE), chromogranin A (CgA), and synaptophysin (SYN) [8,9].

**Figure 2:**
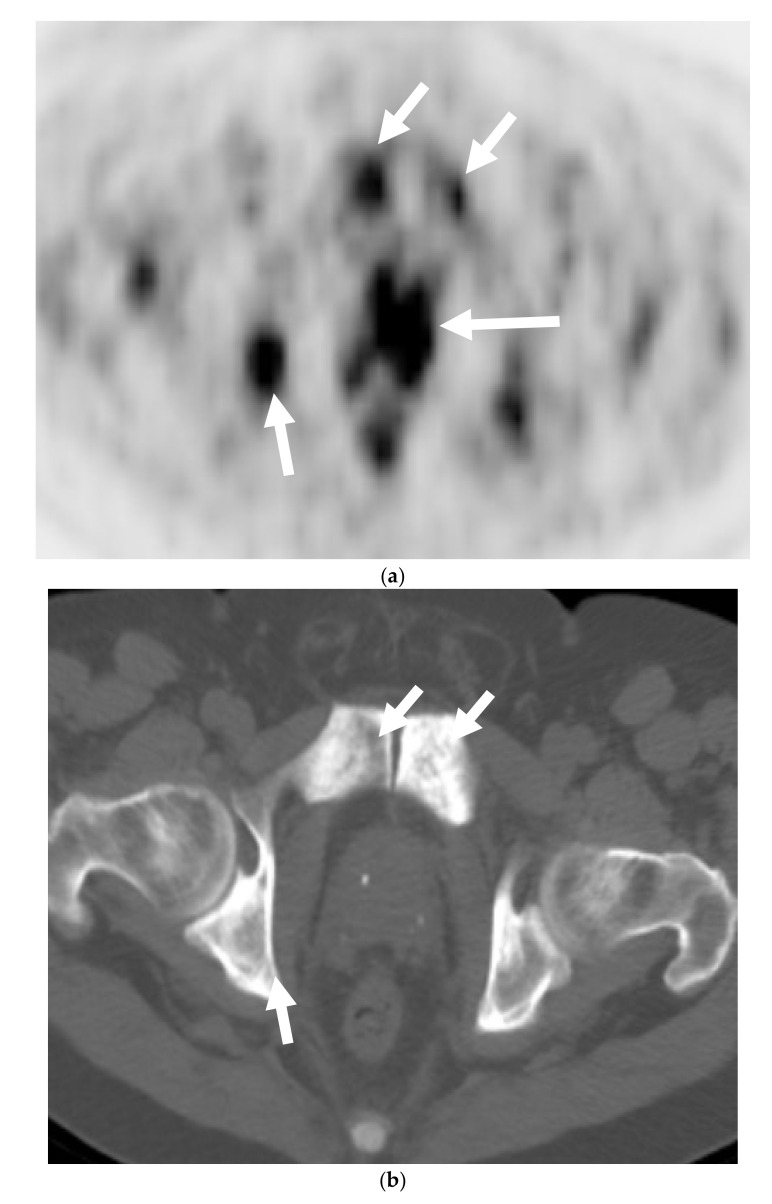

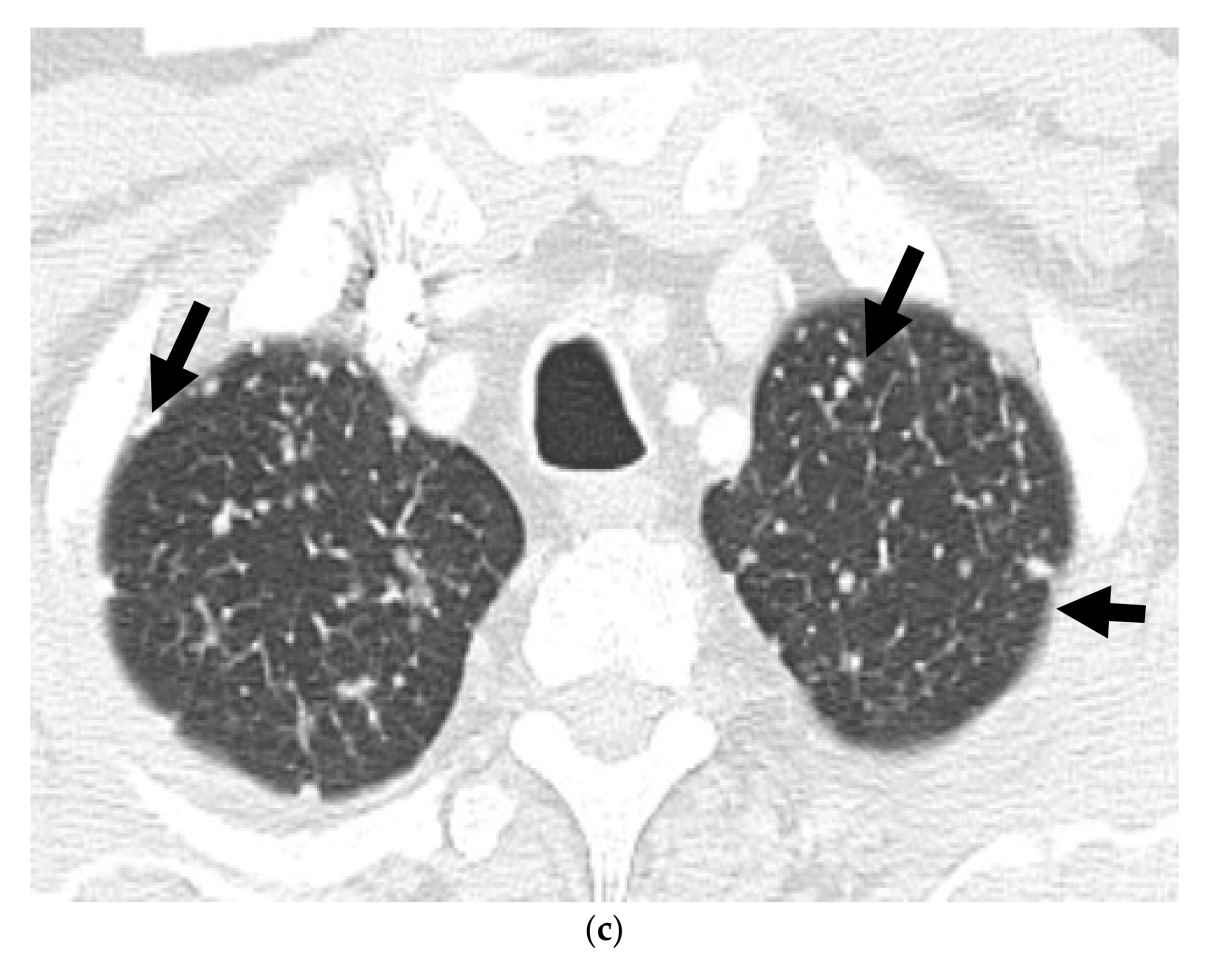
A 68-year-old man with palpable prostatic mass on rectal examination. Serum PSA was 0.3 ng/mL. Axial FDG-PET (**a**) and corresponding anatomic CT (**b**) of the PET-CT shows intensely hypermetabolic prostatic mass (long arrow) and pelvic bone metastases (short arrows). Axial lung window reformat of the CT chest (**c**) shows numerous small pulmonary nodules suspicious for metastases (arrows). Prostate biopsy showed high-grade carcinoma with neuroendocrine differentiation.

According to the most recent classification by the World Health Organization (WHO), NEPCs are classified into 5 categories as follows [7,10]:

1. Usual adenocarcinoma with neuroendocrine differentiation (Figure 2)
  - This type includes cases of typical acinar or ductal adenocarcinoma that have focal neoplastic neuroendocrine cells detected by immunohistochemical stains (IHC). It is subdivided into two subtypes, focal and diffuse [11].
2. Adenocarcinoma with Paneth-like cell neurodifferentiation
  - Defined as a typical adenocarcinoma of the prostate with a varying degree of Paneth-like cells (distinct eosinophilic cytoplasmic granules on LM). It has a favorable prognosis, but it may lead to a false high-grading due to its formation of the nest and cord structures [12].
3. Well-differentiated neuroendocrine tumor (carcinoid tumor)
  - True carcinoid tumor of the prostate is very rare. It has the same morphology of carcinoid tumors elsewhere in the body, including bladder, gastrointestinal tract, and lungs [13]. Diagnosing a carcinoid tumor, especially in a young patient, should raise the clinical suspicion of multiple endocrine neoplasia syndrome, type IIB (MEN IIB) [14].
4. Small cell neuroendocrine carcinoma (SCNC)
  - This is an aggressive, high-grade tumor with identical pathologic features to those of small cell lung carcinoma and other small cell lung cancers. They are typically negative for PSA and androgen receptors, but there are some exceptions as it can be a component of mixed tumor that has the classic luminal adenocarcinoma [15,16].
5. Large cell neuroendocrine carcinoma (LCNC)
  - This newly adopted term describes a high-grade carcinoma that shows neuroendocrine differentiation with large polygonal cells, abundant cytoplasm, and nuclei that contain coarse chromatin and a prominent nucleolus. As a result, it cannot be classified as a SCNC. Pure LCNC is exceedingly rare and most cases occur after long standing hormonal therapy for prostatic adenocarcinoma [17,18].

### 2.1. Genetic Alterations

Identification and understanding of molecular and genetic alterations that lead to neuroendocrine differentiation in prostate cancers is crucial for the development of novel targeted therapies [19]. The most important molecular alterations in applied clinical practice currently are N-myc proto-oncogene (MYCN) and aurora kinase A (AURKA), androgen receptor gene amplification, and ETS gene family fusions. It may also include RE1-silencing transcription factor (REST) gene downregulation or loss, TP54 loss, RB1 loss, PTEN loss, MYCL amplification or upregulation of proliferative genes (e.g., cyclin D1) [20,21,22,23].

### 2.2. Imaging Evaluation

Imaging of the prostate includes various modalities, including multiparametric ultrasound (US), magnetic resonance imaging (MRI), computed tomography (CT), and positron emission technique (PET), including evolving molecular imaging techniques.

Multiparametric US imaging includes various US techniques used for anatomic assessment, such as grayscale US, color doppler US (CDUS), transrectal US (TRUS) biopsy, US elastography (real time and strain), contrast-enhanced US (CEUS), and computer-aided US imaging analysis [24,25]. US imaging for prostate cancer has quite a few drawbacks. For example, benign lesions of the prostate, such as benign prostatic hyperplasia (BPH) and prostatitis, can both have the same hypoechoic appearance of prostate cancer and early-stage cancers can appear isoechoic. Initial TRUS can miss up to 47% of cancer cases and around 60% of suspicious prostatic lesions on grayscale US, are benign [24,26,27]. CEUS has the ability to visualize the asymmetrical tumor microvasculature pattern and makes it superior to CDUS, which is limited to larger macrovessels [28]. As prostate cancers are usually more stiff than normal prostatic tissue due to increased collagen deposition around the tumor, increased cellularity and vascularity, US elastography is emerging as an important diagnostic tool for primary prostatic evaluation [29].

**Figure 3:**
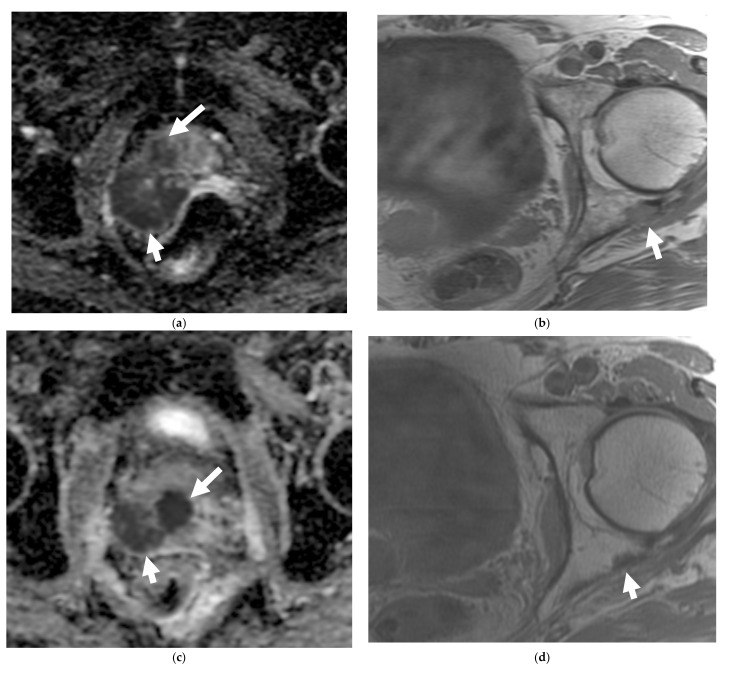

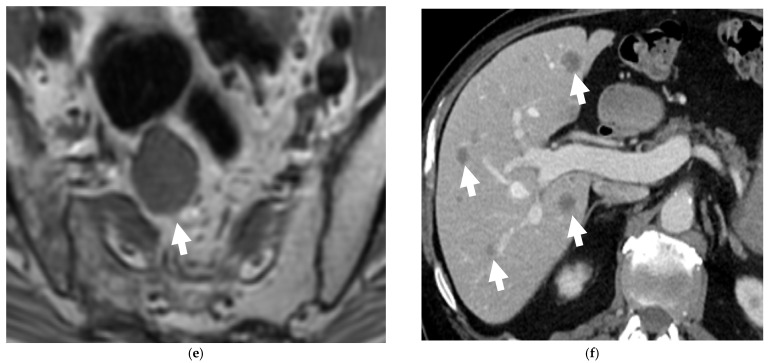
A 60-year-old male with Gleason 9 prostate adenocarcinoma at diagnosis, with later neuroendocrine differentiation after 1 year while on androgen deprivation therapy, with metastatic neuroendocrine prostate cancer. Axial ADC map of the MRI prostate (**a**) shows hypointense right prostatic nodule (with restricted diffusion), consistent with prostatic cancer (long arrow). There is a large mass along the right posterolateral aspect of the prostate, consistent with extraprostatic extension (short arrow). Axial T1W fast spin echo (FSE) image (**b**) shows bony metastasis involving the left posterior acetabulum. Patients’ serum PSA decreased following androgen deprivation therapy. Follow up MRI after 7 months revealed improving (smaller) extraprostatic component of the prostatic mass and left acetabular bony metastasis with relatively unchanged size of the prostatic neoplasm on the apparent diffusion coefficient (ADC) map (**c**) and axial T1W FSE (**d**) images. However, newly enlarged right presacral node (arrow) seen on T1W FSE (**e**) of follow-up MRI pelvis and multiple new liver lesions (arrows) seen on CECT (**f**) at 10 months and 18 months, respectively, after the diagnosis, were biopsied and proven to be metastatic adenocarcinoma with neuroendocrine differentiation.

Most of the literature states that MDCT typically plays no role in the detection of PNEC and is not recommended for diagnosis. The only role of CT is for nodal staging, but it is also limited for this purpose, due to its inability to detect neoplastic architectural changes within less than 10 mm normal-sized lymph nodes (LNs) [30,31]. MDCT plays an important role in M staging for detection and restaging for bone and lung metastases in these cases (Figure 2 and Figure 3).

Multiparametric MRI (mpMRI) is now considered to be the standard imaging evaluation of choice when suspecting prostate cancer. Members of PI-RADS (version 2.1) steering committee recommend using 3T MRI scanners over 1.5T machines for prostatic evaluation, as it increases the signal-to-noise ratio (SNR), leading to an increase in both temporal and spatial resolution. If only 1.5T scanners are available or in the case of inherently low SNR sequences, such as DWI, they recommend the use of endorectal coil (ERC) which has the ability to increase SNR at any magnetic field strength [32]. Most tumors appear isointense to normal prostate tissue on T1-weighted sequences which serve as a baseline for the contrast-enhanced MRI, delineate the prostate outline, and can also demonstrate post-biopsy hemorrhage and periprostatic fat invasion. T2-weighted (T2W) sequences are used to evaluate prostatic zonal anatomy, primarily evaluate the transitional zone or central gland tumors, asses for seminal vesicle or nodal involvement, and detect extra-prostatic extension (EPE). Peripheral zone cancers usually demonstrate ill-defined T2 hypointense focal lesions with restricted diffusion and are primarily evaluated on ADC/DWI images (Figure 1). Transitional zone tumors appear hypointense with spiculated, ill-defined margins and smudgy appearance on T2W images. These lesions may also invade the urethral sphincter and anterior fibromuscular stroma [33,34]. While mpMRI is now considered the technique of choice for initial and local (T) tumor staging, PET/CT and PET/MRI have shown a great value in distant extraprostatic (N and M) staging (Figure 2), restaging after biomedical relapse, and response assessment after androgen deprivation therapy (ADR) [35,36,37,38]. The sensitivity, specificity, positive predictive value, and negative predictive value of multiparametric MRI for detection of EPE (Figure 3), were 48.7%, 73.9%, 35.9%, and 82.8%, respectively [39,40].

mpMRI can also differentiate prostatic carcinoid from usual prostatic adenocarcinoma based on the considerably larger size and mild hyperintensity of the tumor on T2W images [41]. Recently, biopsy guided by the fusion of MRI and transrectal US images (called MRI-TRUS fusion biopsy) is increasingly used where MRI findings are used as reference for US-guided biopsy, allowing for increased accuracy and precision [42].

### 2.3. Molecular Imaging

**Figure 4:**
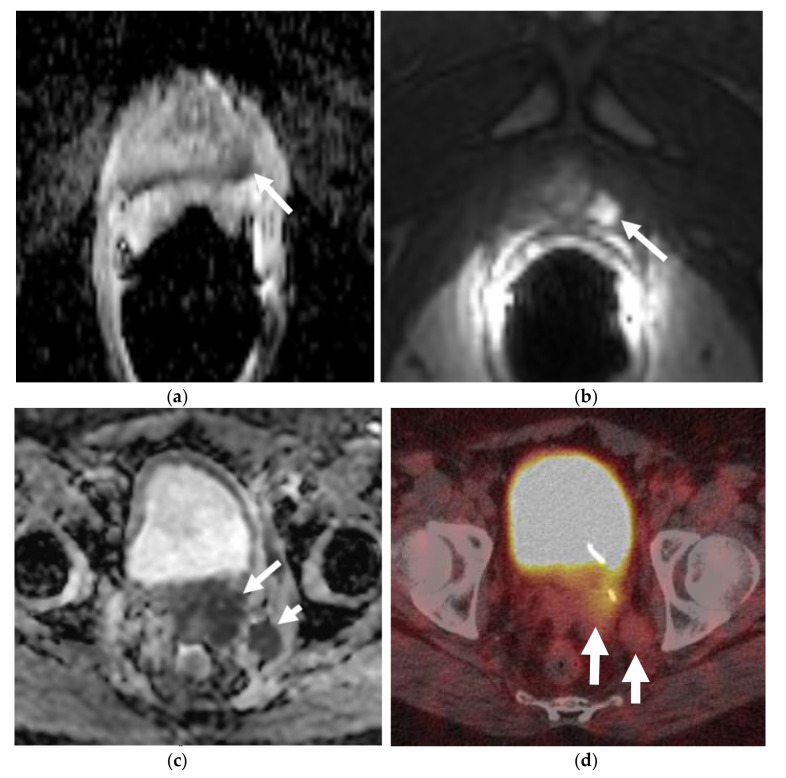
A 56-year-old male with prior hormonal therapy for 2 years and external beam radiation therapy for the biopsy-proven diagnosis of prostatic adenocarcinoma in 2008. Follow-up MRI pelvis performed for recurrent elevated serum PSA 6 years after shows a left posterolateral peripheral zone prostatic nodule with restricted diffusion on ADC map (**a**) and avid early enhancement on the dynamic postcontrast T1W images (**b**), suspicious for recurrent prostatic neoplasm (arrow). This was biopsy proven to be prostatic adenocarcinoma. Further follow-up MRIs after 12 years showed rapidly enlarging left prostatic neoplasm (long arrow) with regional left internal iliac lymphadenopathy (short arrow) on ADC image (**c**) along with metastatic left common iliac and retroperitoneal lymphadenopathy. This was biopsy proven to be metastatic prostatic neuroendocrine carcinoma. Prostatic mass showed heterogeneous hypermetabolism on the axial color fused image of the Ga-68 DOTATATE PET-CT (**d**). The nodal metastases (arrow) were without suspicious hypermetabolism due to poorly differentiated neuroendocrine neoplasm/carcinoma component.

**Figure 5:**
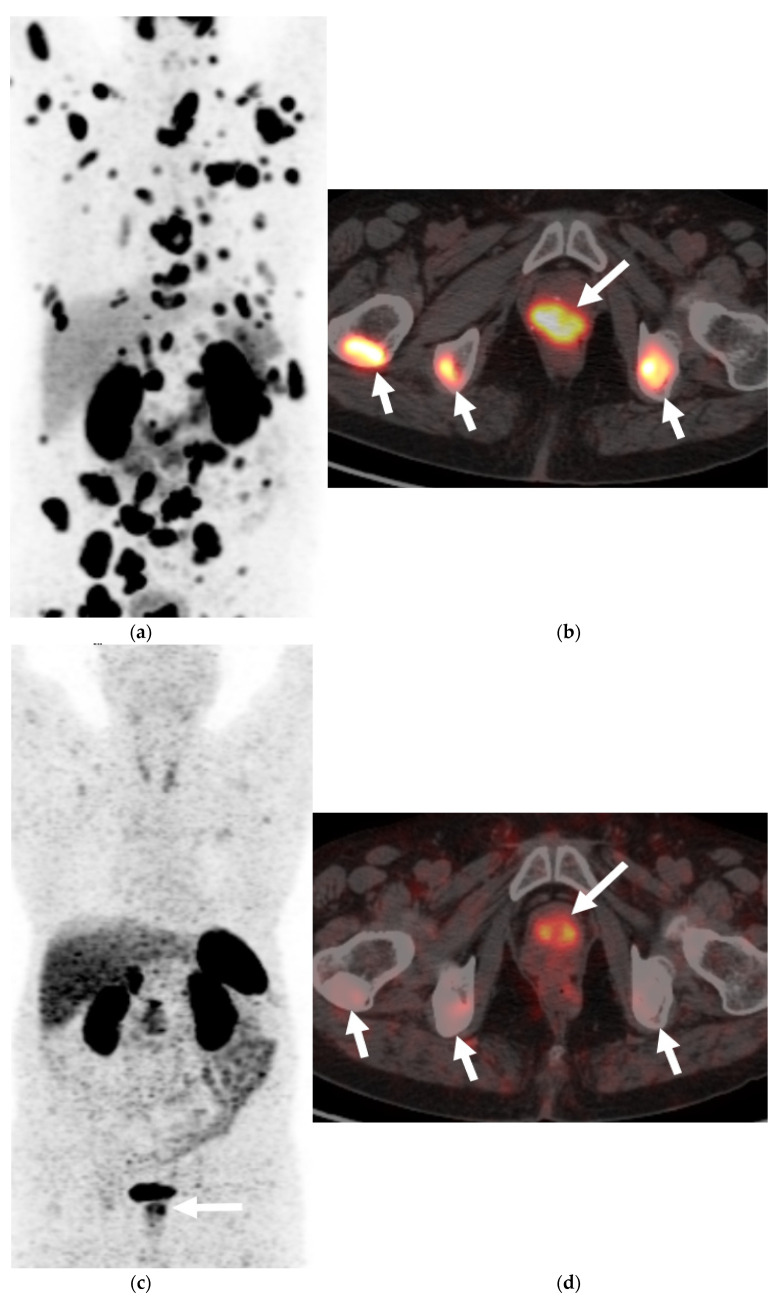
An elderly patient with metastatic neuroendocrine prostate cancer. Coronal maximum intensity projection (MIP) PET image (**a**) and axial color fused image of 68Ga DOTANOC PET-CT (**b**) show intensely hypermetabolic prostatic mass (long arrow) consistent with neuroendocrine prostate cancer and hypermetabolic widespread bony metastases (short arrows). Post-treatment DOTANOC PET-CT following 4 cycles of PRRT therapy shows marked positive treatment response. There was near complete metabolic response in the bony metastases, as seen on the coronal MIP PET image (**c**) with minimal residual metabolic activity corresponding to some sclerotic bony metastases on axial color fused PET-CT image (**d**). There is also interval decrease in the extent and degree of hypermetabolic activity in the prostatic malignancy with residual activity suspicious for residual viable malignancy. Image courtesy: Divya Yadav, M.D.

Molecular imaging in prostate cancer offers the advantage of improved sensitivity over conventional imaging. Multiple PET tracers are available for the evaluation of prostate carcinoma, particularly in the restaging setting. The FDA-approved radiotracers include 18F-FDG, 18F-NaF, 11C/18F-Choline, and 18F-Fluciclovine. 68Ga-DOTATATE PET (Figure 4 and Figure 5) has been found to be promising and is now being established for the evaluation of neuroendocrine neoplasms of the lungs, thyroid gland, and gastrointestinal tract. However, it is still not used for routine clinical use in patients with NEPC [43,44]. PSMA has received attention as a useful biomarker in the imaging of prostate cancer, particularly detecting disease at lower PSA levels. However, the expression may be reduced that can potentially lead to false negatives in highly evolved tumors with neuroendocrine features [45]. Another emerging PET tracer is an analog of bombesin or antagonist of the gastrin releasing peptide receptor. Bombesin-like peptides are also overexpressed in NEPC and are an area of active research [46].

#### 2.3.1. 18 F- Fluorodeoxyglucose (FDG)

FDG is a glucose analog and its uptake reflects the tissue glucose metabolism. Due to increased uptake in neoplasms, resulting from the increased metabolic activity of the tumor cells, it is the mainstay of molecular imaging and the most commonly used PET tracer to evaluate tumors [47,48]. It has a limited value when it comes to prostate cancer as a result of low glucose metabolism and the use of non-glucose metabolic pathways, e.g., fructose and fatty acid metabolism in the tumor [49,50]. However, Spratt et al. demonstrated that 18FDG PET has clinical utility in the metastatic evaluation of NEPC (Figure 2) and this may be due to high glucose metabolism of the usually high-grade neuroendocrine cancers seen in prostate. FDG PET findings can also serve as prognostic marker in cases of metastatic NEPC. When stratified by the median survival from NEPC diagnosis, patients who survived <2.2 versus ≥2.2 years, had more PET avid bone and soft tissue lesions and higher average SUVmax of bone and soft tissue lesions [51,52]. Some low-grade neuroendocrine tumors may not be intensely FDG-avid and rather may be more intensely avid on 68 Gallium DOTATATE PET, as shown with gastroenteropancreatic neuroendocrine neoplasms [53].

#### 2.3.2. 68 Gallium Labelled Somatostatin Analogs (^68^Ga-DOTATATE or ^68^Ga-DOTANOC)

^68^Ga-DOTATATE or *^68^Ga-DOTANOC*, are *^68^Ga* labeled somatostatin analogs that bind with high affinity to the somatostatin receptor 2 (SSTR2), which is highly expressed by NEPCs, enabling their identification by SSTR2 tracers [54,55]. 68Ga-DOTATATE or *^68^Ga-DOTANOC* PET can be used to evaluate bony metastases and predict treatment response in these cases [54,56]. 68Ga-DOTATATE has a reported sensitivity and specificity of 82% and 90%, respectively, for detecting disease in cases of biochemically-relapsed prostate cancer [57]. This may be presumably useful in evaluation and management of low and intermediate grade neuroendocrine neoplasms, as shown in cases of gastroenteropancreatic neuroendocrine neoplasms [53,58,59]. At the same time, one should also remain aware of the false positive diagnosis in the setting of prostatitis due to inflammatory uptake [60,61] or in case of standard prostatic adenocarcinoma with inflammatory cell infiltrates [62]. Inflammatory tracer uptake usually gives rise to low- or very low-grade hypermetabolic activity and may be a clue in some of these cases [60].

## 3. Discussion

Neuroendocrine tumors are a heterogeneous group of malignancies originating from neuroendocrine cells, which are either embedded in endocrine organs or dispersed throughout the body. Such cells have dense-core secretory granules with the ability to secrete bioactive peptides, e.g., neurotransmitters, neuromodulators, or neuropeptide hormones. They are defined immunohistochemically by their markers, such as chromogranin A (CgA), neuron-specific enolase (NSE), and synaptophysin (SYN) [63]. These also serve as general serum tumor markers for screening patients for neuroendocrine neoplasms in cases without distinct hormone-related clinical features [63,64]. Among these, CgA has been found to be more sensitive and specific for various neuroendocrine neoplasms [63,64], it correlates with tumor bulk, and may also predict treatment response [64].

In prostate, neuroendocrine cells typically constitute a minority (<1% of cells) of the gland. Cancers arising from these cells are some of the most aggressive prostate cancers. In contrast to the most common cell subtype of prostate adenocarcinoma, which has cells morphologically similar to the luminal prostate cells, are androgen dependent and typically associated with elevated serum PSA; NEPCs are androgen independent and not associated with elevated serum PSA. Rather, they express and secrete neuroendocrine markers, including NSE, CgA, and synaptophysis (SYN), in the serum [8,9]. Focal neuroendocrine differentiation, which is driven by androgen deprivation therapy (ADT) and also termed as therapy-induced NEPC (t-NEPC), has been reported in 17–30% of prostate adenocarcinoma cases within approximately 5 years of follow-up during treatment [15,19,65]. Neuroendocrine differentiation in prostate cancer is frequently associated with advanced stage disease, impaired quality of life, and poor prognosis [66]. Adenocarcinoma with NE differentiation, as well as SCNC and LCNC, are considered more aggressive tumors with rapid progression, worse prognosis, and drastically reduced cancer-specific survival (median cancer-specific survival is less than 2 years) [67,68,69].

There is no core difference in staging between prostatic adenocarcinoma and NEPCs. The TNM staging system, developed by the American Joint Committee on Cancer (AJCC), is the most commonly used staging method to assess the tumor status (T), lymph nodes (N), and metastasis (M) [70]. Unlike prostatic adenocarcinoma, clinical tumor stage 1 is uncommonly seen in cases with NEPC and they usually present with higher stages, with more visceral and nodal metastases with predominantly lytic bone lesions. Hence, NEPC is clinically suspected when a prostate cancer is seen with absent or a low/moderate rise in PSA, presents at advanced stage, or has a predominance of visceral and/or bone metastatic disease (Figure 2). In addition, NEPC is also suspected when the prostate cancer becomes unresponsive to ADT with rapid disease worsening (Figure 3 and Figure 4) [68,69]. Paraneoplastic syndrome is also a potential distinguishing feature for NEPCs, especially SCNC, with Cushing’s syndrome being the most common manifestation [71,72]. Currently, the reference standard for the diagnosis of NEPCs is pathologic examination showing the above microscopic features, plus the presence of neuroendocrine IHC markers, e.g., NSE, SYN, and CgA [16,73].

From an imaging point of view, the most commonly employed imaging modalities for prostate biopsies and cancer detections are multiparametric ultrasound (US) and magnetic resonance imaging (MRI). Computed tomography (CT) is reserved for staging purposes. Thus far, conventional imaging methods cannot directly differentiate between NEPCs and prostatic adenocarcinoma; however, the presence of nodal and visceral metastases and rapid progression may suggest NEPC, which often requires histopathologic confirmation [74,75]. Various PET tracers are now available for the imaging of prostate cancer and neuroendocrine differentiation. A recent clinical case series demonstrated excellent detection rates for the metastases (95%), specifically the visceral and nodal metastases in NEPCs by using FDG-PET [51,52]. The positron-emitting somatostatin analogs, which bind to somatostatin receptors (SSRTs) with high affinity, 68 Ga-DOTATATE or 68 Ga DOTANOC are also suitable and found to be as highly accurate as the PET/CT radiotracers, for the display of somatostatin-expressing NETs [43,44]. Usmani et al. reported a case that showed the significance of somatostatin receptor scintigraphy for the detection of neuroendocrine differentiation of metastatic prostate cancer [76]. In another study of 12 patients with metastatic NEPC previously treated with ADT, 50% of the patients showed a moderate or high tracer uptake in the metastases on the 68 Ga-DOTATATE-PET/CT. Further research with higher numbers of cases is required to assess the performance of 68 Ga-Somatostatin analog-PET/CT as a new diagnostic tool for prostatic neuroendocrine neoplasms [55].

The role of artificial intelligence is also being investigated to improve the differentiating ability of imaging modalities for different subtypes of prostate cancer in the future. In a recent study by Lal et al., different machine learning techniques, e.g., support vector machine (SVM) kernels, radial base function (RBF), and Gaussian and decision tree, with different feature-extracting strategies, such as scale invariant feature transform (SIFT), and elliptic Fourier descriptors (EFDs) features, were utilized for detecting different types of prostate cancers [77]. Circulating tumor cells (CTCs) from patients with NEPC have unique morphologic characteristics which can also be very important for diagnosis in the near future, as well as assessing prognosis as the CTC count correlates inversely with NEPC prognosis [73,78].

Similar to most other neuroendocrine tumors, such as small-cell lung cancer, NEPC tends to be sensitive to chemotherapy and radiotherapy. Currently, treatment of NEPCs consists mainly of prostatectomy with adjuvant platinum-based regimens. Cisplatin/carboplatin combinations with either docetaxel or etoposide, have relatively high response rates. The addition of doxorubicin to regimens has not demonstrated advantages and is associated with increased side effects [79,80,81]. In a recent study, Apostolidis et al. concluded that NEPCs can be treated similar to NETs of the gastrointestinal tract, with somatostatin analogs, everolimus, and peptide receptor radionuclide therapy (PRRT) (Figure 5) [82]. Adjuvant radiotherapy is also very important, particularly when positive surgical margins are identified [80]. Targeted therapy is an exciting new therapy for NEPCs, as early evidence suggests improved clinical outcomes. Alisertib, an agent that can inhibit the interaction between N-myc and its stabilizing factor Aurora-A, has recently entered into a phase II clinical trial. It inhibits N-myc signaling, which is a driver of NEPC progression and thus, suppresses tumor growth [83,84].

## 4. Conclusions

NEPC is a highly lethal subtype of prostate cancer with higher metastatic potential and poor survival in comparison to the most common type of prostate cancer, prostate adenocarcinoma. Rapid worsening of prostate cancer, low serum PSA despite advanced prostate cancer, predominance of visceral (particularly liver) and osteolytic bone metastases, should lead one to suspect NE differentiation of prostate cancer either de novo or as CRPC. TRUS biopsy, multiparametric MRI, and MRI-TRUS fusion biopsy have increasing roles in imaging assessment and disease management. Functional and molecular imaging, as well as machine learning techniques, appear promising in the diagnosis of NEPC and its distinction from prostate adenocarcinoma and should be further investigated. Therefore, elucidating the role of genetic alterations for targeted therapeutics holds promise for improved diagnosis and management of patients suffering from aggressive NEPC.

## Data Availability

No new data were created or analyzed in this study. Data sharing is not applicable to this article.
